# Safety and efficacy outcomes after intranasal administration of neural stem cells in cerebral palsy: a randomized phase 1/2 controlled trial

**DOI:** 10.1186/s13287-022-03234-y

**Published:** 2023-02-09

**Authors:** Zhongyue Lv, Ying Li, Yachen Wang, Fengyu Cong, Xiaoyan Li, Wanming Cui, Chao Han, Yushan Wei, Xiaojun Hong, Yong Liu, Luyi Ma, Yang Jiao, Chi Zhang, Huanjie Li, Mingyan Jin, Liang Wang, Shiwei Ni, Jing Liu

**Affiliations:** 1 Stem Cell Clinical Research Center, The First Affiliated Hospital of Dalian Medical University, No. 193, Lianhe Road, Shahekou District, Dalian, 116011 Liaoning China; 2 Dalian Innovation Institute of Stem Cell and Precision Medicine, Dalian, Liaoning China; 3 School of Biomedical Engineering, Faculty of Electronic Information and Electrical Engineering, Dalian University of Technology, Dalian, Liaoning Province, China; 4 Faculty of Information Technology, University of Jyvaskyla, 40014 Jyvaskyla, Finland; 5 Department of Ent, The First Affiliated Hospital of Dalian Medical University, Dalian, Liaoning China; 6 Scientific Research Department, The First Affiliated Hospital of Dalian Medical University, Dalian, Liaoning China; 7 Neurophysiological Center, The First Affiliated Hospital of Dalian Medical University, Dalian, Liaoning China; 8 Department of Rehabilitation, The First Affiliated Hospital of Dalian Medical University, Dalian, Liaoning China; 9 Department of Pediatrics, The First Affiliated Hospital of Dalian Medical University, Dalian, Liaoning China; 10 Department of Neurology, The First Affiliated Hospital of Dalian Medical University, Dalian, Liaoning China; 11 School of Biomedical Engineering, Dalian University of Technology, Dalian, Liaoning China

**Keywords:** Neural stem cells, Cerebral palsy, Intranasal administration, Electroencephalogram, Clinical trials, Functional brain network

## Abstract

**Background:**

Neural stem cells (NSCs) are believed to have the most therapeutic potential for neurological disorders because they can differentiate into various neurons and glial cells. This research evaluated the safety and efficacy of intranasal administration of NSCs in children with cerebral palsy (CP). The functional brain network (FBN) analysis based on electroencephalogram (EEG) and voxel-based morphometry (VBM) analysis based on T1-weighted images were performed to evaluate functional and structural changes in the brain.

**Methods:**

A total of 25 CP patients aged 3–12 years were randomly assigned to the treatment group (*n* = 15), which received an intranasal infusion of NSCs loaded with nasal patches and rehabilitation therapy, or the control group (*n* = 10) received rehabilitation therapy only. The primary endpoints were the safety (assessed by the incidence of adverse events (AEs), laboratory and imaging examinations) and the changes in the Gross Motor Function Measure-88 (GMFM-88), the Activities of Daily Living (ADL) scale, the Sleep Disturbance Scale for Children (SDSC), and some adapted scales. The secondary endpoints were the FBN and VBM analysis.

**Results:**

There were only four AEs happened during the 24-month follow-up period. There was no significant difference in the laboratory examinations before and after treatment, and the magnetic resonance imaging showed no abnormal nasal and intracranial masses. Compared to the control group, patients in the treatment group showed apparent improvements in GMFM-88 and ADL 24 months after treatment. Compared with the baseline, the scale scores of the Fine Motor Function, Sociability, Life Adaptability, Expressive Ability, GMFM-88, and ADL increased significantly in the treatment group 24 months after treatment, while the SDSC score decreased considerably. Compared with baseline, the FBN analysis showed a substantial decrease in brain network energy, and the VBM analysis showed a significant increase in gray matter volume in the treatment group after NSCs treatment.

**Conclusions:**

Our results showed that intranasal administration of NSCs was well-tolerated and potentially beneficial in children with CP.

*Trial registration*: The study was registered in ClinicalTrials.gov (NCT03005249, registered 29 December 2016, https://www.clinicaltrials.gov/ct2/show/NCT03005249) and the Medical Research Registration Information System (CMR-20161129-1003).

**Supplementary Information:**

The online version contains supplementary material available at 10.1186/s13287-022-03234-y.

## Introduction

Cerebral palsy (CP) is the leading cause of physical disability in children [1, 2], which is a group of non-progressive permanent central nervous system (CNS) diseases that affect the motor, postural, and coordination functions [3]. The global prevalence of CP was around 2.0–3.5 per 1000 live births [4, 5], and among them, 80% of cases are classified as spastic [6]. The electroencephalography (EEG) and neuroimaging abnormalities have been observed in most CP patients [7]. Recently, stem cell therapy has shown favorable function in repairing nerve damage in CP [8, 9]. Some stem cell therapies have been trialed in patients with CP [10–12] and have improved functional recovery. However, many aspects of stem cell therapy remain unknown, such as the best route of cells administration, the most appropriate type of stem cells, the accurate assessments of clinical efficacy, and the neuropathological mechanism of stem cell therapy in humans [13].

Several stem cell transplantation schemes have been applied in CP patients, like intrathecal administration (brain stereotactic surgery, lumbar puncture) and intravenous/intraarterial injection. Still, they reflect some drawbacks, such as procedure-related adverse events (AEs) [13]. A new and better therapeutic strategy with well-tolerated, high efficacy, and few AEs will maximize the therapeutic effect of stem cells for children with CP. Preclinical studies show that intranasal administration of stem cells for neurological disorders is a promising cellular delivery method and can reduce the incidence of AEs in patients [14]. Neural stem cells (NSCs) are believed to have the most therapeutic potential for neurological disorders because they can differentiate into various neurons and glial cells. In hypoxia–ischemia neonatal models, intranasal delivery of NSCs improved brain injury and neurological outcomes [15]. A clinical observation reported that the brain injury area of preterm infants with intracranial hemorrhage reduced significantly after receiving a nasal drip of breast milk (including nutritional factors and breast milk stem cells), which further supported the feasibility of intranasal delivery of stem cells [16]. However, the clinical trial of intranasal administration of stem cells in treating neurological disease has not been reported.

As we all know, motor function deficits occur as a result of brain function and structure injury. Although clinical scale evaluation could intuitively reflect the improvements of some symptoms, it is difficult to provide direct evidence for the alterations in brain function and structure. EEG is a commonly used method to evaluate brain function in the clinic. The sleep EEG was a reliable index of the brain structural and functional changes that could reflect the brain cortical reorganization and synapses pruning of neural activity in children and adolescents [17, 18]. However, there is no way for clinicians to assess brain function by quantitatively analyzing EEG data. Neurons in the brain form a complex network. Graph theory-based functional brain network (FBN) provides a new perspective for EEG quantitative analysis [19]. The FBN has important topological properties, such as degree, global efficiency, clustering coefficient, characteristic path length, and brain network energy, which provides a new method to quantify brain function [20]. And this helps to explain the topological principle of functional reconstruction of the nervous system and brain [21, 22] and may be a new promising method for analyzing brain function changes in CP patients after stem cell therapy. Magnetic resonance imaging (MRI) is the most commonly used technology to reveal structural brain changes in the clinic. We all know that ischemia and hypoxia are the major contributor to CP [23] and could lead to neuronal and glial injury. Previously, the research group used the diffusion tensor imaging (DTI) sequence of MRI to evaluate the effect of stem cell therapy on the integrity of white matter (the primary concentration area of glial cells). However, it is unknown whether stem cell therapy affects the changes of neurons in the human body. The survival and functional recovery of neurons are crucial for functional neurological repair. The soma of neurons is located in the gray matter. The morphometric changes of the brain are conventionally tested using voxel-based morphometry (VBM). T1-weighted image analysis of MRI may provide reliable evidence for the recovery of neurons and provide a reference for studying the neuropathological mechanism of stem cell therapy in the human body.

Herein, we conducted an open-label randomized controlled clinical trial to evaluate the safety and efficacy of intranasal administration of NSCs for CP for the first time. And we performed FBN analysis based on sleep EEG and VBM analysis based on T1-weighted images before and after treatment to evaluate the effects of NSCs on brain function and gray matter structure.

## Materials and methods

### Study design

This phase 1/2, randomized, controlled clinical trial was designed to evaluate the safety and efficacy of intranasal delivery of NSCs loaded by degradable patches. According to the principle of continuous enrollment, we randomly assigned all 25 patients who met the enrollment conditions from January 2018 to March 2019, including 15 patients in the treatment group (receiving NSCs transplantation three times and rehabilitation therapy; Patients 1–15) and 10 patients in the control group (receiving rehabilitation therapy only; Patients 16–25). This study was registered with ClinicalTrials.gov (NCT03005249) and the Medical Research Registration Information System (CMR-20161129–1003). And it was approved by the Ethics Committee of the First Affiliated Hospital of Dalian Medical University. The reference number of the ethics approval was LCKY2016-60.

### Eligibility and screening

Eligible participants, aged between 3 and 12 years, were clinically presenting with moderate to severe paralysis characterized by spastic CP induced by ischemic hypoxia. The Gross Motor Function Classification System (GMFCS) levels were II-V (Patient recruitment conditions are shown in Additional file 1). All the subjects had participated in regular rehabilitation training for no less than one year but had no apparent improvements in clinical symptoms 3–6 months before treatment. All the participants’ guardians signed informed consent before participating in the study.

### The NSCs nasal patch complex preparation

The allogeneic NSCs were isolated from aborted human fetal forebrain tissue. These tissues were collected from healthy pregnant women who had requested an artificial abortion. All the donors voluntarily signed informed consent forms, and the tests for human immunodeficiency virus, syphilis, hepatitis B, and other pathogenic microorganisms were negative. All donor tissue was stored in sealed containers of sterile 0.9% normal saline (NS) and transported to the stem cell preparation center registered with the China Food and Drug Administration (CFDA) via a cold chain within 6 h of abortion. The whole procedure was in accordance with the current international Good Manufacturing Practices process for separation, extraction, amplification, passage, and detection. The NSCs were passaged every 7–10 days and carried out using passages 4–8 for our clinical research. Before clinical application, cytopathic tests were performed for endotoxins, mycoplasma, chlamydia, and viruses. And the proportions of positive antigens expression were calculated by Flow Cytometry assay of NSCs biomarkers (Vimentin, Nestin, Notch-1, SOX2, SSEA1and Musashi-1). Immunofluorescence staining was used to detect the differentiation ability of NSCs into neurons, astrocytes, and oligodendrocytes. Observation of interaction between patches and NSCs was performed by overlaying images of cells and materials and then analyzed by confocal photographing. 2 × 10^6^ cells/mL of NSCs were seeded at the materials and cultured in a 37 °C incubator with 5% CO_2_ atmosphere for 21 days to test the degradation of patch materials in vitro by measuring the dry weight of cells and patch complexes. The 100%, 50%, 25%, and 0% extract of patch materials were used to culture NSCs for 14 days to test the biocompatibility of NSCs and materials, and CCK-8 tests were used to detect the cytotoxicity of patch materials.

### The NSCs nasal patch complex transplantation

Patients in the treatment group took oral *Loratadine* tablets (5 mg) and received intravenous *Dexamethasone* (4 mg) 2 h before transplantation. And then, they were sedated with 10% *Chloral Hydrate* enema (50 mg/kg). NSCs (5 × 10^5^/kg) loaded with patches were placed on the olfactory fissure in bilateral nasal cavities by an otolaryngologist within 5 min. After that, patients were required to lay supine and monitored for at least 4 h. Every child received NSCs transplantation three times with an interval of 1 month.

### Safety assessments

The short-term safety profile was tested for one week after NSCs transplantation, including AEs, physical examinations, daily vital signs, dietary sleep records, physical examination records of pediatric neurologists and rehabilitation practitioners (blind method), blood biochemical examinations, ultrasonic cardiography, radiographic images of the chest and articulation/coxae, electrocardiogram, EEG, and MRI. And the long-term safety profile was monitored up to 24 months after NSCs infusion.

### Scales analysis

The clinical symptoms analysis includes the gross motor, fine motor, sleep quality, social ability, speech, life adaptability, and self-care ability. The scales used for measurements include the Gross Motor Function Measure-88 (GMFM-88), the Activities of Daily Living (ADL) scale, the Sleep Disturbance Scale for Children (SDSC), and some adapted scales derived from the Gesell Growth Scale and the Chinese Psychological Development Scale, like the Fine Motor Function Scale (FMFS), the Sociability Scale (SS), the Life Adaptability Scale (LAS), and the Expressive Ability Scale (EAS). All scales were evaluated by neurologists and pediatricians blinded to the whole study. And the data were collected at baseline (1 week before the treatment), 1 month (1 M), 3 months (3 M), 6 months (6 M), and 24 months (24 M) after NSCs treatment for all the CP patients.

### EEG acquisition and preprocessing

The sleep EEG data were collected by a Micromed Brain SPY Plus (Micromed, Italy) at baseline, 6 M, and 24 M after NSCs treatment for all the CP patients. EEG signals were recorded at a 128 Hz sampling rate according to the international 10–20 placement system at the following positions: C3, C4, P3, P4, O1, O2, P7, and P8. The reference electrode was located at Cz, and the ground electrode was located at the central forehead. Electrode impedance was kept below 10 kΩ. Electrocardiogram data were recorded in a separate channel. Ten segments of EEG data in the non-rapid eye movement phase (NREM) II stage of sleep were randomly intercepted by an experienced technician, and each segment lasted 15 s. EEG signal was high pass-filtered at 0.30 Hz and low pass-filtered at 30 Hz.

### Functional brain network construction

FBN refers to a graph that includes the nodes and functional connections formed by the cooperation and coordination of neural activities between neurons and between parts of the nervous system. The most commonly used method to construct FBN is the Pearson correlation coefficient (PCC) (the EEG data processing flow is shown in Additional file 2). The Degree, Clustering coefficient, Characteristic path length, Global efficiency, and Brain network energy analysis were performed to compare the difference with the baseline or the control group (see Additional file 3 for the analysis methods).

### MRI acquisition

Data were acquired from each subject with a 3.0 T GE Signa HDxt scanner, including T1-weighted imaging and T2-weighted imaging. Three-dimensional high-resolution T1 weighted images were scanned by brain volume scanning sequence (BRAVO) with the following parameters: TR = 8.2 ms, TE = 3.2 ms, TI = 450 ms, FA = 12°, FOV = 256 mm × 256 mm, acquisition matrix = 256 × 256, layer thickness = 1 mm, and the number of layers = 188. Patients with cognitive impairment were examined under sedation with 10% chloral hydrate.

### Voxel-based morphological analysis

VBM analysis was performed with FSL software, which can quantitatively calculate local gray matter voxels' size and signal intensity. Skull stripping was performed first, and then, a gray matter template was created at a resolution of 2 × 2 × 2 mm^3^ in standard space. And then, transform each voxel’s gray matter density values into gray matter volume values, followed by nonlinear registration. Use Gaussian smoothing kernel and image to discrete convolution operation, and perform the spatial smoothing of gray matter volume map after registration transformation (the result of 8 mm smoothing kernel is selected for the following analysis in this analysis). Finally, the results of different smoothing degrees are obtained, that is, each subject’s gray matter volume map.

### Statistical analysis

All statistical analyses were performed with the Software Package for Social Sciences for Windows v23.0 (IBM, Armonk, New York). Categorical data are presented as frequencies and percentages. Continuous data are presented as the mean ± standard deviation (SD) or median (Q1-Q3) as appropriate. To compare categorical data between groups, *χ*^*2*^ tests were performed. Continuous data were compared using the independent Student’s *t* test or Mann–Whitney *U* test, as appropriate. Changes of variables at 24 months relative to baseline in the study and control group were compared using paired Student’s *t* test or Wilcoxon rank-sum test. Changes in variables at baseline, 1 M, 3 M, 6 M, and 24 M in the study group were compared using the one-way repeated-measures ANOVA or generalized linear mixed model. A two-tailed *P* value < 0.05 was considered statistically significant.

## Results

### Patients

**Fig. 1:**
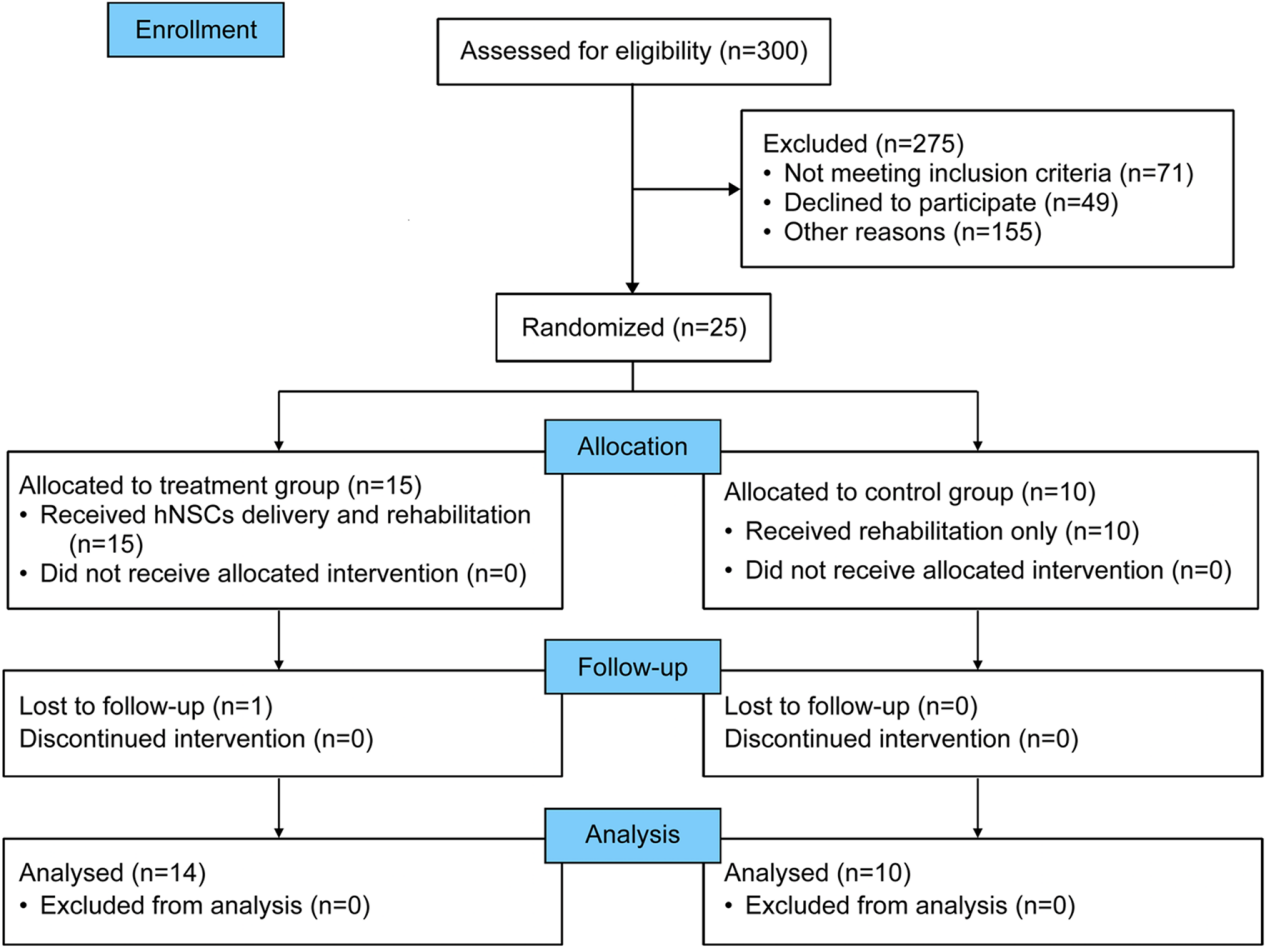
Consolidated Standards of Reporting Trials (CONSORT) flow diagram

Figure 1 summarizes the numbers of patients screened, enrolled, and excluded. Of the 300 eligible patients, 275 were excluded due to severe comorbidities or unwillingness to participate. A total of 25 patients were enrolled (further information is provided in Additional file 4), of whom 15 (6.73 ± 2.12 years old) were randomized to the treatment group and 10 (5.72 ± 1.97 years old) to the control group. There were no significant differences between the two groups of patients in terms of baseline characteristics, including age, sex, body weight, GMFCS, and the Manual Ability Classification System (MACS) (Table 1). Routine management was provided to both groups of patients after initial hospitalization and perioperatively. Six patients had mild anemia before treatment and had been given iron supplementation throughout the treatment. The last patient completed 24 months of follow-up in August 2021. Finally, 24 patients were included in the statistical analysis, and one patient was lost to follow-up.

**Table 1:** Baseline demographics and characteristics

	Treatment group (*n*, %)	Control group (*n*, %)	*P* value
*Age, years*			
Mean ± SD	6.73 ± 2.12	5.72 ± 1.97	0.249
> 6 years	8 (57%)	5 (50%)	
≤ 6 years	6 (43%)	5 (50%)	
*Sex*			1.000
Male	10 (71%)	7 (70%)	
Female	4 (29%)	3 (30%)	
Body weight (kg)	18.82 ± 4.13	22.10 ± 7.62	0.187
*GMFCS*			0.783
II	3(21%)	3(30%)	
III	4(29%)	3(30%)	
IV	5(36%)	4(40%)	
V	2(14%)	1(10%)	
*MACS*			0.162
II	5(36%)	6(60%)	
III	3(21%)	1(10%)	
IV	4(29%)	1(10%)	
V	2(14%)	2(20%)	

*SD*: standard deviation; *kg*: kilogram; *GMFCS*: gross motor function classification system; *MACS*: manual ability classification system

### The NSCs nasal patch complex

**Fig. 2:**
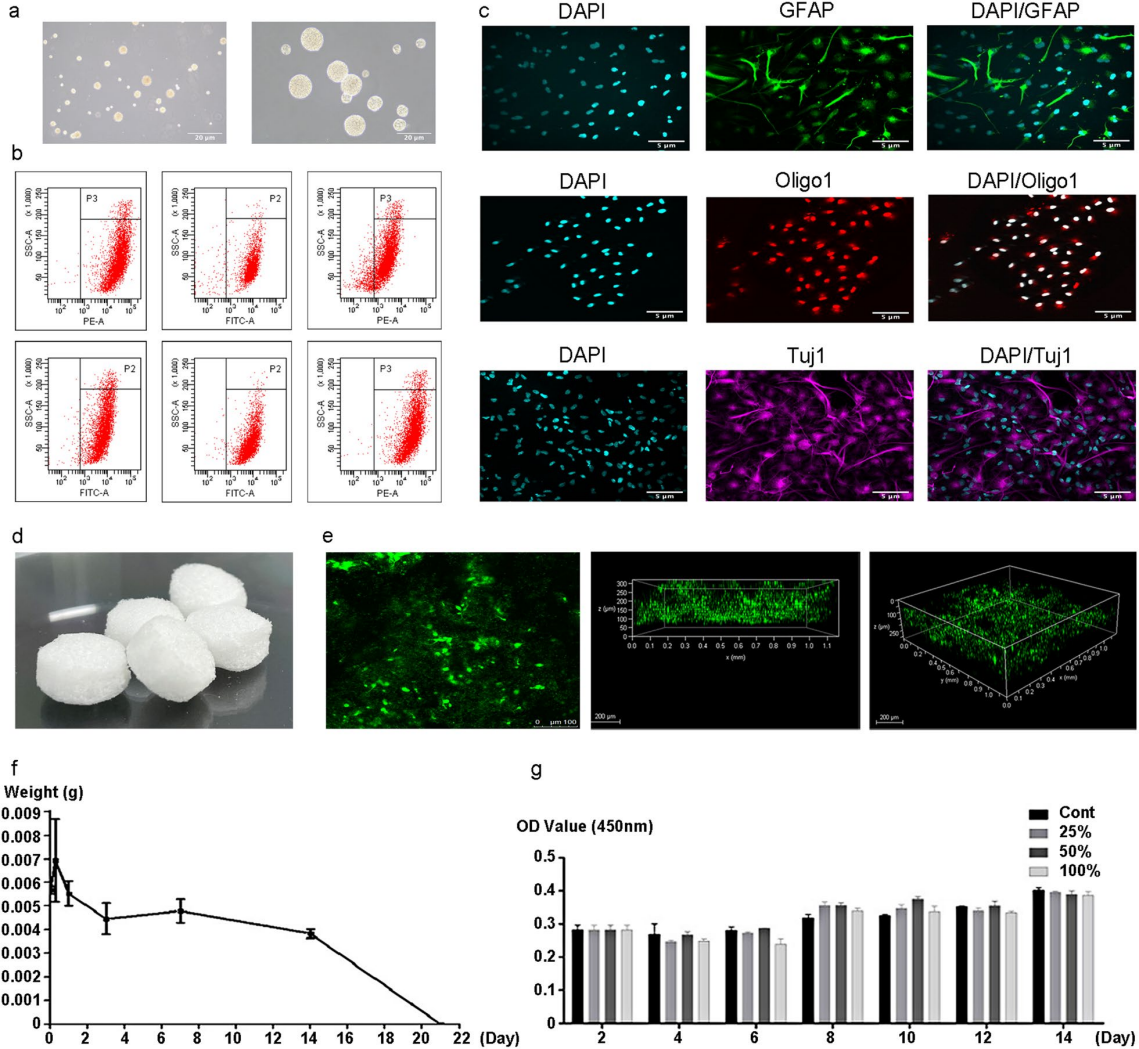
The NSCs Nasal Patch Complex. **a**: Neurospheres of human NSCs (P6) cultured in vitro under the microscope; **b**: The identification of human NSCs by Flow cytometry: Vimentin, Nestin, Notch-1, Sox2, SSEA1, and Musashi-1; **c**: Differentiation ability of NSCs into neurons (Tuj1), astrocytes (GFAP) and oligodendrocytes (Oligo1); **d**: Physical drawing of the nasal patch after magnification of 5 × ; **e**: Observation of NSCs nasal patch complexes by laser confocal microscope; **f**: NSCs nasal patch complex biodegradation weight-time curve in vitro: at 21d, the samples were gelatinous, all dissolved after washing to achieve complete degradation; **g**: The growth curve of NSCs cultured in material extracts of different concentrations (cont, 25%, 50%, 100%) was obtained from the CCK-8 test results: Cont: control

The NSCs grew spherical in vitro (Fig. 2a), and the flow cytometry analysis showed that NSCs biomarkers were positive (Fig. 2b). When cultured in vitro, NSCs can differentiate into neurons, oligodendrocytes, and astrocytes (Fig. 2c). The biodegradable patch materials loaded with NSCs, which have filed a patent application (International Patent Number: PCT/CN2019/077105; Chinese patent number: PCT/CN2019/077105; 201,810,623,850.5), were made of gelatin sponge and cut into cylinders with a diameter of 8 mm and a thickness of 5 mm (Fig. 2d). Confocal immunofluorescence microscopy showed that NSCs were uniformly distributed within the patch, as shown in Fig. 2e. The test of degradability of patches before treatment showed that patches degraded utterly at 21 days in vitro (Fig. 2f). The cytotoxic assay showed that the 100%, 50%, 25%, and 0% extract of path materials had no toxicity to NSCs growth (Fig. 2g).

### The primary endpoints

#### Adverse events

All 25 patients received the intended treatment of either NSCs combined with rehabilitation or rehabilitation alone. No severe AEs related to the intranasal delivery treatments or NSCs occurred 24 months after transplantation. Most of the observed AEs were grade I and II in severity or were transient, lasting a few hours or up to 3 days following transplantation (Table 2). Only four AEs happened during the 24 months follow-up period in the treatment group. Two patients had a low-grade fever (body temperature < 38℃) but recovered without medicine. One patient experienced nasal mucosa hemorrhage (volume < 0.1 mL) following nasal delivery and recovered following oppression hemostasis. One patient had a history of complex partial seizure (CPS) and took oral antiepileptic drugs before treatment. Three months after NSCs treatment, the patient’s CPS type and frequency changed and were controlled by adjusting the dose of sodium valproate and increasing carbamazepine.

**Table 2:** Adverse events of safety outcomes between groups

	Treatment group	Control group
*Severe adverse events*		
Seizure	1	0
Urinary tract infection	0	0
Pneumonia	0	0
Influenza	0	0
Death	0	0
*Other adverse events*		
Otitis media acute	0	0
Nasal mucosa hemorrhage	1	0
Conjunctival irritations	0	0
Constipation	0	0
Dyspepsia	0	0
Nausea, vomiting	0	0
Diarrhea	0	0
Colitis	0	0
Anorexia	0	0
Herpangina	0	0
Fever	2	0
Febrile convulsion	0	0
Irritability	0	0
Insomnia	0	0
Bronchitis	0	0
Hypoxia	0	0
Dermatitis	0	0
Urticaria	0	0
Hirsutism	0	0
Alopecia	0	0

#### Laboratory and imaging examinations

There were no significant differences in laboratory tests between baseline and after the whole course of treatment in the treatment group (Additional file 5). MRI of the nasal cavity and brain did not reveal any noticeable pathological changes in any patient, including microbleeds, mass formation associated with cell transplantation, infection, or tumor formation. Neither of the two groups showed any evidence of an increase in systemic inflammation or other immune responses.

#### Scales analysis

One patient was lost to follow-up in the treatment group because of the local COVID-19. Twenty-four patients completed the follow-up during 24 months of the clinical research. We evaluated clinical improvements among the treatment and control groups using scale data at baseline and the endpoint of 24 months. The scale scores in gross motor function, fine motor function, self-care ability, sociability, expressive ability, and sleep quality showed no apparent differences between the treatment and control groups at baseline (Table 3).

**Table 3:** Clinical changes of scales between groups

	Treatment group	Control group	*P* value
*FMFS*			
Baseline	24.00 ± 15.85	26.50 ± 14.99	0.701
24 M	36.56 ± 17.86^#^	24.56 ± 14.25^#^	0.136
*SS*			
Baseline	25.50(8.75–45.00)	26.00(15.13–46.75)	0.546
24 M	48.50(23–53.50)^#^	27.50(13.75–44.63)^#^	0.238
*LAS*			
Baseline	23.50(11.13–50.63)	31.75(13.50–48.25)	0.841
24 M	54.00(22.50–56.00)^#^	27.25(15.00–51.88)^#^	0.238
*EAS*			
Baseline	17.50(8.00–51.75)	29.25(10.75–50.50)	0.886
24 M	54.00(19.00–56.00)^#^	34.00(11.75–52.63)^#^	0.395
*GMFM-88-A (Lying and Rolling)*			
Baseline	51(44.5–51)	49(38.5–49.5)	0.259
24 M	51(51–51)	48(36.5–50.5)	0.041^*^
*GMFM-88-B (Sitting)*			
Baseline	44.5(26.25–57)	46.5(29–56.5)	0.796
24 M	59(56–60)^#^	41.5(21.5–57.75)	0.051
*GMFM-88-C (Crawling and Kneeling)*			
Baseline	18.5(6.75–33)	7(0–35.25)	0.625
24 M	40(35–42^)#^	5(0–32.25)	0.016^*^
*GMFM-88-D (Standing)*			
Baseline	17(7.5–23.5)	7.5(0–18.5)	0.172
24 M	27(25–31)^#^	3.5(0–17)	0.002^*^
*GMFM-88-E (Walking, Running and Jumping)*			
Baseline	11(2–25.25)	4.5(0–16.25)	0.285
24 M	31(12–47)^#^	1.5(0–12)	0.007^*^
*GMFM-88-TOTAL*			
Baseline	52.41 ± 24.44	47.43 ± 27.30	0.644
24 M	74.95 ± 23.84^#^	41.94 ± 24.50	0.009^*^
*ADL*			
Baseline	34.54 ± 20.90	40.65 ± 31.72	0.574
24 M	61.68 ± 26.16^#^	32.81 ± 20.09	0.018^*^
*SDSC*			
Baseline	49.43 ± 14.27	47.90 ± 19.81	0.828
24 M	39.14 ± 9.77^#^	47.50 ± 18.54	0.164

Data are summarized as the mean ± SD (standard deviation). GMFM: Gross Motor Function Measure; ADL: Activity of Daily Living; SDSC: Sleep Disturbance Scale for Children; FMFS: Fine Motor Function Scale; SS: Sociability Scale; LAS: Life Adaptability Scale; EAS: Expressive Ability Scale; 24 M: 24 months after treatment

*P < 0.05 (compared with Control group); #*P* < 0.05 (compared with Baseline)

As shown in Table 3, compared with the control group, patients in the treatment group had obvious improvements in the self-care ability and gross motor function evaluated by the ADL and GMFM-88 (including A, C, D, E, and TOTAL) scales at 24 M after NSCs administration (*P* < 0.05). The score of the FMFS, SS, LAS, EAS, and GMFM-88-B showed a tendency toward an increase, and SDSC showed a trend to decrease in the treatment group, although not statistically significant. Compared with the baseline, the scale scores of the FMFS, SS, LAS, EAS, GMFM-88 (including B, C, D, E, and TOTAL), and ADL increased significantly in the treatment group at 24 M, while the SDSC score decreased (*P* < 0.05). But in the control group, GMFM-88, ADL, and SDSC showed no statistically changes, and the FMFS and LAS decreased statistically significantly (*P* < 0.05). The scores of the SS and EAS showed obviously increased but were much less pronounced than the treatment group. These results suggested that intranasal administration of NSCs could improve the patient's clinical symptoms such as movement, language, cognition, self-care ability, and even sleep quality.

To explore the onset time of NSCs in improving the clinical symptoms of patients with CP and the stability of its therapeutic effect, we further analyzed the evaluation scale data of patients in the treatment group at different time points. We observed a statistically significant increase in all scales except for LAS and GMFM-88-A at different follow-up time points (Table 4 and Additional file 6). Patients' activities of self-care ability (ADL, from 34.54 ± 20.90 to 45.85 ± 24.34, *P* < 0.01), standing (GMFM-88-D, from 15.86 ± 10.33 to 22.85 ± 11.51, *P* < 0.05), and walking/running/jumping (GMFM-88-E, from 14.29 ± 13.60 to 21.77 ± 15.24, *P* < 0.01) began to show significant improvement as early as 1 M after treatment. 3 M after treatment, expressive ability (EAS, from 29.29 ± 21.41 to 36.92 ± 19.86, *P* < 0.05) and the overall function of gross motor (GMFM-88-TOTAL, from 52.41 ± 24.44 to 71.45 ± 22.29, *P* < 0.05) had been significantly improved. 6 M after treatment, the patients' social skills (SS, from 26.68 ± 18.65 to 32.82 ± 17.82, *P* < 0.05) improved, and other previous improvements were maintained. The improvement of the most complex hand fine motor, which requires high control of small muscles, muscle tone, and hand–eye coordination, did not begin until 24 M (FMFS, from 24.00 ± 15.85 to 36.56 ± 17.86, *P* < 0.05). And all the significant improvements persisted until 24 M post-transplantation in the treatment group. The results suggested that NSCs intranasal administration could result in long-term improvements for CP patients and the effects were more pronounced at the early stage of gross motor.

**Table 4:** Changes of scales from baseline to month 24 in the treatment group

	Baseline	1 M	3 M	6 M	24 M	*P* value
FMFS	24.00 ± 15.85	28.42 ± 16.57	29.33 ± 15.68	31.54 ± 17.93	36.56 ± 17.86*	.010*
SS	26.68 ± 18.65	30.58 ± 18.54	34.17 ± 17.40	32.82 ± 17.82*	39.00 ± 16.73**	.000**
LAS	29.36 ± 21.00	32.12 ± 20.65	33.46 ± 18.63	33.32 ± 19.87	39.23 ± 19.27	.205
EAS	29.29 ± 21.41	33.31 ± 21.21	36.92 ± 19.86*	33.86 ± 20.08*	39.14 ± 19.36*	.003*
GMFM-88-A (Lying and Rolling)	45.36 ± 11.53	47.77 ± 9.98	47.33 ± 10.33	48.29 ± 8.82	47.82 ± 9.91	.084
GMFM-88-B (Sitting)	39.29 ± 20.57	49.85 ± 19.27	52.58 ± 16.05	50.29 ± 19.08	53.36 ± 16.82	.040*
GMFM-88-C (Crawling and Kneeling)	19.79 ± 14.29	28.46 ± 14.56	31.33 ± 12.15	27.29 ± 13.76	35.18 ± 12.17	.014*
GMFM-88-D (Standing)	15.86 ± 10.33	22.85 ± 11.51*	25.92 ± 9.42*	23.14 ± 11.79	25.36 ± 10.39**	.001**
GMFM-88-E (Walking, Running and Jumping)	14.29 ± 13.60	21.77 ± 15.24**	25.75 ± 17.13**	26.07 ± 19.21**	31.18 ± 18.79**	.000**
GMFM-88-TOTAL	52.41 ± 24.44	66.66 ± 25.02	71.45 ± 22.29*	67.95 ± 24.72*	74.95 ± 23.84*	.001**
ADL	34.54 ± 20.90	45.85 ± 24.34**	52.08 ± 24.38**	50.82 ± 27.00**	61.68 ± 26.16**	.000**

Data are summarized as the mean ± SD (standard deviation). 1 M: 1 month after treatment; 3 M: 3 months after treatment; 6 M: 6 months after treatment; 24 M: 24 months after treatment; GMFM: Gross Motor Function Measure; ADL: Activity of Daily Living; FMFS: Fine Motor Function Scale; SS: Sociability Scale; LAS: Life Adaptability Scale; EAS: Expressive Ability Scale

*P < 0.05; **P < 0.01

### The secondary endpoints

#### Functional brain networks of EEG

**Fig. 3:**
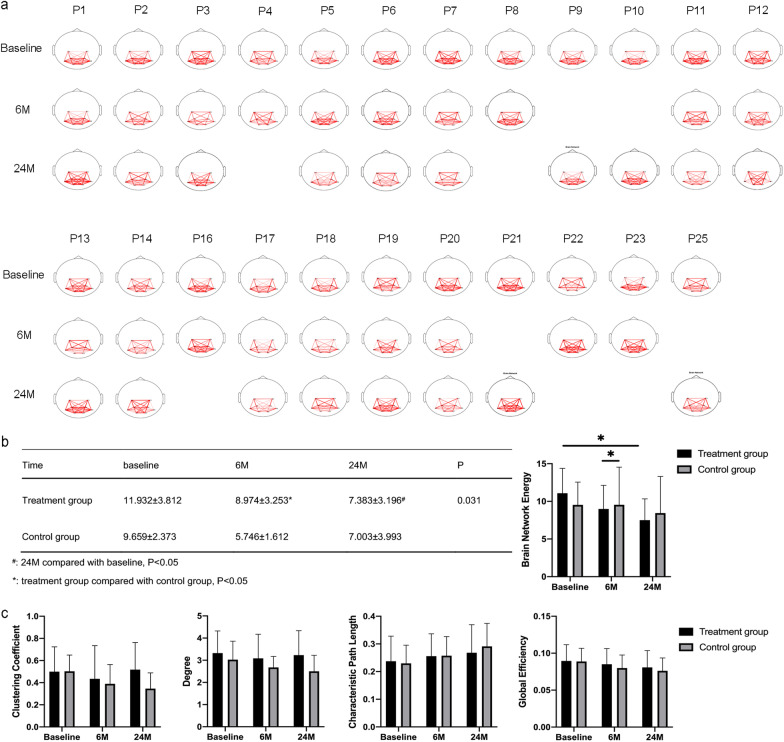
Brain functional network analysis. **a**: The functional brain network in sleep period of all patients; **b**: Changes of brain network energy in two groups; **c**: Changes of degree, clustering coefficient, global efficiency, and characteristic path length in two groups; Baseline: before treatment; 6 M: 6 months after treatment; 24 M: 24 months after treatment; P1-P14: patients in the treatment group; P16-P25: patients in the control group; Node: the locations of the eight electrodes (C3, C4, P3, P4, O1, O2, P7, P8); Red line: the connectivity between nodes, the thicker the red line, the stronger the correlation, and the thinner the red line, the weaker the correlation

To further explore the changes in brain function in CP patients subjected to NSCs transplantation, we performed a graph theory-based FBN analysis of EEG in all enrolled patients. As shown in Fig. 3a, the FBN connection showed a gradual weakening trend in sleep EEG in both groups with the progress of the intervention. Then, we calculated five topological features of FBN based on graph theory, including the degree (*D*), clustering coefficient (*C*), characteristic path length (*L*), global efficiency (*E*), and brain network energy (BNE) [24, 25]. The results showed that there was a significant difference (*P* < 0.05) in the BNE (Fig. 3b) between two groups 6 M after NSCs treatment, and the *D*, *C*, and *E* showed an upward trend, and the *L* showed a downward trend, but no statistical difference between the two groups (Fig. 3c). There was no interaction between time and grouping. Compared with baseline, the BNE in the treatment group showed a significant decrease in 24 M (*P* < 0.05), while it also decreased in the control group, but there was no significant difference. In the control group, the *D*, *C*, and *E* showed a downward trend along with time, but in the treatment group, the *D* and *C* showed a *U*-shaped curve, while the *E* showed a downward trend. The *L* showed an upward trend in both groups but no significant difference. The decrease in BNE might suggest an increase in sleep homeostasis in all the patients, especially in the children who received NSCs transplantation.

#### VBM analysis of T1-weighted images

VBM analysis was carried out 6 M after NSCs treatment to determine changes in the volume of the cerebral cortex. Owing to some patients' T1-weighted image artifacts, we only performed VBM analysis on the T1-weighted images of nine patients in the treatment group. We found that the overall brain volume of CP patients showed an increasing trend after treatment, with an increase of 1.51%, although there was no significant difference compared with baseline. Further analysis in local brain regions indicated that the gray matter related to the patient's visual, language, sensory, hand–eye coordination, and social and emotional processing significantly increased after NSCs treatment, such as superior occipital gyrus, middle occipital gyrus, left inferior occipital gyrus, right inferior occipital gyrus, fusiform gyrus, superior parietal gyrus, inferior parietal lobe, angular gyrus, and temporal pole middle temporal gyrus (*P* < 0.05) (the results are shown in Table 5).

**Table 5:** Changes of gray matter volume from baseline to month 6 in the treatment group

	Baseline	6 M	*P* value
Total volume	614,239.25 ± 39,964.24	623,498.50 ± 40,216.82	0.438
49 (SOG.L)	4924.49 ± 685.36	5164.16 ± 729.92	0.035*
52 (MOG.R)	8141.31 ± 1263.95	8737.89 ± 1148.10	0.019*
53 (IOG.L)	4474.26 ± 485.94	4860.14 ± 544.49	0.042*
54 (IOG.R)	4239.01 ± 797.49	4680.28 ± 814.30	0.023*
56 (FFG.R)	11,843.16 ± 1078.77	12,450.73 ± 960.36	0.008**
60 (SPG.R)	5877.22 ± 960.71	6482.12 ± 926.72	0.005**
62 (IPL.R)	5238.69 ± 587.67	5713.49 ± 566.96	0.007**
66 (ANG.R)	6505.39 ± 727.61	7008.39 ± 871.93	0.043*
87 (TPOmid.L)	2390.20 ± 654.50	2586.36 ± 506.35	0.031*

6 M: 6 months after treatment; SOG.L: Superior occipital gyrus; MOG.R: Middle occipital gyrus; IOG.L: Inferior occipital gyrus; IOG.R: Inferior occipital gyrus; FFG.R: Fusiform gyrus; SPG.R: Superior parietal gyrus; IPL.R: Inferior parietal, but supramarginal and angular gyri; ANG.R: Angular gyrus; TPOmid.L: Temporal pole: middle temporal gyrus

*P < 0.05; **P < 0.01

## Discussion

This study is a randomized controlled clinical trial with a small sample size and evaluates the safety and efficacy of intranasal delivery of NSCs composite patches for the treatment of CP. No severe AEs associated with NSCs treatment were observed throughout the treatment duration and follow-up. The clinical assessment of scales showed that NSCs intranasal administration could improve the gross motor, fine motor, self-care ability, expressive ability, social skills, and sleep quality of CP patients. The data from EEG and MRI provide evidence for brain functional and structural improvements after NSCs treatment.

The most suitable administration route of stem cells is still unknown. Intrathecal administration and intravenous/intraarterial injection are the most commonly used delivery strategies for stem cell therapy in CP. Many AEs (such as bleeding at the puncture site, infection, and cerebrospinal fluid leakage) caused by the intrathecal injection may reduce the surgical tolerance of CP children and the secondary injury introduced by the transplantation method neutralizes part of the effects mediated by stem cells. During intravascular infusion, stem cells tend to accumulate in organs with rich capillary networks owing to the large volume [26, 27], especially the pulmonary and liver, increasing the possibility of microvascular embolism. Intranasal administration decreases AEs incidence and severity. The number of AEs during the whole treatment period was 4 in our study, much lower than 49 AEs in 14 patients with intravenous injection [10]. Mild AEs, such as low fever and nasal mucosal bleeding, were effectively controlled by simple physical therapy. One boy had a history of seizures and took oral antiepileptic drugs (sodium valproate and levetiracetam). He had no grand mal one year before treatment but CPS once a week which showed disturbance of consciousness with the rigidity of the right upper limb. 3 M after NSCs treatment, CPS type changed and was always induced by intense light stimulation without consciousness disturbance. Through clinical laboratory testing, we found that the blood concentration of sodium valproate decreased due to the patient weight increased by 10 kg. Finally, the boy’s CPS was reduced to once biweekly by adjusting the dose of sodium valproate and increasing carbamazepine for treatment. We believed that the decrease in CPS frequency and the disappearance of consciousness disorder during CPS might be related to NSCs treatment.

Meanwhile, the limitation of BBB makes it difficult for stem cells to enter the central nervous system through intravascular infusion, reducing the utilization rate of stem cells [28]. Invasive manipulation like intrathecal administration is poorly tolerated by children. Intranasal administration provides a non-invasive method of bypassing the BBB to potentially effectively deliver stem cells to the brain [29] to avoid secondary damage to brain tissue caused by the invasive operation [14, 30]. In our studies, patients in the treatment group showed noticeable gross motor and self-care ability improvements compared with the control group. And compared with baseline, patients showed significant improvement in gross motor, fine motor, self-care ability, expressive ability, social skills, and sleep quality after NSCs therapy. (The videos for comparisons of symptom improvements before and after treatment are shown in Additional files 7, 8, 9, 10, 11, 12). The functional improvements were maintained for 24 months after NSCs treatment and continue to improve. The scale evaluation of patients in the treatment group at different time points after NSCs treatment shows that the notable improvements in clinical symptoms appeared as early as 1 M after NSCs transplantation and are stable over time. These clinical results provide strong evidence for the nasal administration of NSCs in the treatment of CP from clinical trial to clinical application.

The detection of FBN is helpful in measuring the recovery of brain function. We all know that the normal operation of brain networks requires neuronal activity with plasticity and stability [31]. Following nerve injury, functional neural circuits were impaired due to the axons, dendrites, and synapses of neurons eliminated [32]. The implantation of stem cells promotes nerve proliferation, neuron growth, and synapse formation and establishes a wide range of nerve connections early. Over time, to form more accurate and mature neural circuits, regression events such as axon pruning and synaptic elimination are necessary to remodel functional neural circuits [33, 34]. The researchers have proposed that the recovery of synaptic plasticity mainly occurs during sleep. The sleep quality assessment of CP patients showed significant improvement after NSCs treatment, which suggested increasing sleep homeostasis. Sleep homeostasis is associated with many physiological functions, such as energy metabolism and neural plasticity [35]. During sleep, spontaneous activity consumes a lot of energy to reorganize neural networks, increase synaptic strength and restore cell homeostasis [36]. The decline of topological feature BNE of FBN constructed from sleep EEG data in the treatment group might be related to this kind of regression events-synaptic plasticity remodeling. NSCs can specifically differentiate into various nerve cells and even participate in the remodeling of brain pericytes and neovascularization to build a new neural synaptic network and improve synaptic and neuronal plasticity [37]. The increase in synaptic plasticity is conducive to optimizing the neural network and the maturity of brain function. It is crucial for stem cell-based therapies to restore functional recovery [38]. Our study shows that NSCs intranasal delivery plays a positive role in promoting the regulation of plasticity and stability, and the reduction in BNE may be an important indicator of the function improvement of the cerebral network in patients with CP.

Another manifestation of recovery from nerve injury is morphological reconstruction [39]. Through the VBM analysis of T1-weighted images, we found that 6 M after NSCs treatment, the overall volume of the cerebral gray matter showed an upward trend with an increase of about 1.51% (although not statistically significant). In another study, researchers measured brain morphology from infancy to late adulthood and found that the volume of cortical gray matter peaked around the age of 4 and began to decline after that [40]. In our study, the average age of patients in the treatment group was 6.73 ± 2.12 years old, which indicated that the increase in cortical brain volume did not come from the patient's own growth and development. Further, the increase in brain regions associated with visual, language, sensory, hand–eye coordination, and social and emotional processing provides objective evidence for the morphological effects of neural stem cells on these brain regions.

This study is the first attempt at nasal administration of stem cells in the human body, which provides a reference basis for future nasal transplantation of stem cells. At the same time, we first introduced the FBN analysis based on graph theory to evaluate brain function changes after stem cell therapy in terms of sleep EEG, which provides objective evidence for assessing of the efficacy of stem cell therapy. There were also some limitations exist for this study. First, the open-label design might be a possible limitation for this explorative study. But it is unlikely to affect AEs detection and efficacy assessment as intranasal administration is a minimally invasive operation and the efficacy assessment was regulated to be conducted by independent physicians other than the treating physician. Second, further study should include a placebo-controlled group based on a large-scale double-blinded, randomized controlled trial. Third, extending the follow-up time to obtain long-term safety and efficacy data. Finally, using stem cell tracers and other imaging data, such as magnetic resonance spectroscopy, can help better understand the potential mechanism of stem cells in neuronal repair.

## Conclusions

This study shows that intranasal administration of NSCs was well-tolerated and potentially beneficial in children with CP.

## Supplementary Information

**Additional file 1**. Study inclusion and exclusion criteria. GMFCS: Gross Motor Function Classification System; AIDS: Acquired Immune Deficiency Syndrome; MRI: Magnetic Resonance Imaging.

**Additional file 2**. The EEG data processing flow. Red line: the Connectivity between Nodes; Node: the Locations of the 8 Electrodes (C3, C4, P3, P4, O1, O2, P7, P8).

**Additional file 3**. Functional brain network analysis methods.

**Additional file 4**. Basic information of all the patients. P1-P15: Patients in the treatment group; P16-P25: Patients in the control group; CP: Cerebral palsy; GMFCS: Gross Motor Function Classification System; MACS: Manual Ability Classification System; M: Male; F: Female; SP(q): Spastic (quadriplegic); MT (s+d): Mixed-type (spastic + dyskinetic); MT(s+a): Mixed-type (spastic + ataxic); +: With Disorder; -: Without Disorder; HI: Hypoxic-ischemic; VLBW: Very Low Birth Weight (<1500g); LBW: Low Birth Weight (<2500 g); NW: Normal Weight; GTCS: Generalized Tonic-clonic Seizure; CPS: Complex Partial Seizures.

**Additional file 5**. Comparison of routine biochemical blood parameters before and after stem cell therapy of patients in the treatment group. Data are summarized as the mean±SD (standard deviation). PT: Prothrombin Time; INR: International Normalized Ratio; PTA: Prothrombin Activity; APTT: Activated Partial Thromboplastin Time; TT: Thrombin Time; FIB: Fibrinogen; WBC: White Blood Cell; NEUT: Neutrophil Ratio; LYMPH: Lymphocyte Ratio; MONO: Monocyte; EO: Eosinophilic Granulocyte; BASO: Basophil; RBC: Red Blood Ccell; HB: Hemoglobin; HCT: Hematocrit; MCV: Mean Corpuscular Volume; MCH: Mean Corpuscular Hemoglobin; MCHC: Mean Corpuscular Hemoglobin Concentration; RDW-SD: Red Blood Cell Distribution Width; PLT: Platelets; ALT: Glutamic-pyruvic Transaminase; AST: Glutamic Oxalacetic Transaminase; Cre: Ereatinine; UA: Uric Acid; ALB: Albumin; ALP: Alkaline Phosphatase; γ-GT: γ-Glutamyl Transpeptidase; Na: Sodium; K: Potassium; C1q: Serum Complement C1q; NSE: Neuron Specific Enolase.

**Additional file 6**. Changes of scales from baseline to month 24 between groups. 1M: 1 month after treatment; 3M: 3 months after treatment; 6M: 6 months after treatment; 24M: 24 months after treatment; GMFM: Gross Motor Function Measure; ADL: Activity of Daily Living; FMFS: Fine Motor Function Scale; SS: Sociability Scale; LAS: Life Adaptability Scale; EAS: Expressive Ability Scale; *: P<0.05.

**Additional file 7**. Walking ability of P13 before stem cell therapy.

**Additional file 8**. Walking ability of P13 after stem cell therapy.

**Additional file 9**. Writing ability of P2 before stem cell therapy.

**Additional file 10**. Writing ability of P2 after stem cell therapy.

**Additional file 11**. Speaking ability of P1 before stem cell therapy.

**Additional file 12**. Speaking ability of P1 after stem cell therapy.

## Data Availability

The datasets generated and/or analyzed during the current study are not publicly available due to some data involving patient privacy but are available from the corresponding author on reasonable request.

## Abbreviations

ADL: Activity of daily living
AEs: Adverse events
BNE: Brain network energy
BRAVO: Brain volume scanning sequence
*C*: Clustering coefficient
CFDA: China Food and Drug Administration
CNS: Central nervous system
CP: Cerebral palsy
CPS: Complex partial seizure
*D*: The degree
DTI: Diffusion tensor imaging
*E*: Global efficiency
EAS: Expressive ability scale
EEG: Electroencephalography
FBN: Functional brain network
FMFS: Fine Motor Function Scale
GMFCS: Gross Motor Function Classification System
GMFM-88: Gross Motor Function Measure-88
*L*: Characteristic path length
LAS: Life adaptability scale
MACS: Manual Ability Classification System
MRI: Magnetic resonance imaging
NREM: Non-rapid eye movement phase
NS: Normal saline
NSCs: Neural stem cells
PCC: Pearson correlation coefficient
SD: Standard deviation
SDSC: The Sleep Disturbance Scale for Children
SS: Sociability scale
VBM: Voxel-based morphological
1 M: 1 Month
3 M: 3 Months
6 M: 6 Months
24 M: 24 Months

## References

[1] Wu CL (2020). A pilot study of two different constraint-induced movement therapy interventions in children with hemiplegic cerebral palsy after botulinum toxin injection during preschool education. Front Pediatr.

[2] Kowalski JL (2019). Motor evoked potentials as potential biomarkers of early atypical corticospinal tract development in infants with perinatal stroke. J Clin Med.

[3] Hayashi-Kurahashi N (2012). EEG for predicting early neurodevelopment in preterm infants: an observational cohort study. Pediatrics.

[4] Graham HK (2016). Cerebral palsy. Nat Rev Dis Primers.

[5] Colver A, Fairhurst C, Pharoah PO (2014). Cerebral palsy. Lancet.

[6] Yan Y (2019). Hip adductor intramuscular nerve distribution pattern of children: a guide for BTX-A treatment to muscle spasticity in cerebral palsy. Front Neurol.

[7] Aisen ML (2011). Cerebral palsy: clinical care and neurological rehabilitation. Lancet Neurol.

[8] Chen G (2013). Neural stem cell-like cells derived from autologous bone mesenchymal stem cells for the treatment of patients with cerebral palsy. J Transl Med.

[9] He S (2012). Ultrasound guided neural stem cell transplantation through the lateral ventricle for treatment of cerebral palsy in children. Neural Regen Res.

[10] Sun JM (2021). Sibling umbilical cord blood infusion is safe in young children with cerebral palsy. Stem Cells Transl Med.

[11] Min K (2013). Umbilical cord blood therapy potentiated with erythropoietin for children with cerebral palsy: a double-blind, randomized, placebo-controlled trial. Stem Cells.

[12] Amanat M (2021). Clinical and imaging outcomes after intrathecal injection of umbilical cord tissue mesenchymal stem cells in cerebral palsy: a randomized double-blind sham-controlled clinical trial. Stem Cell Res Ther.

[13] Lv ZY, Li Y, Liu J (2021). Progress in clinical trials of stem cell therapy for cerebral palsy. Neural Regen Res.

[14] Danielyan L (2011). Therapeutic efficacy of intranasally delivered mesenchymal stem cells in a rat model of Parkinson disease. Rejuvenation Res.

[15] Ji G (2015). NF-kappaB signaling is involved in the effects of intranasally engrafted human neural stem cells on neurofunctional improvements in neonatal rat hypoxic-ischemic encephalopathy. CNS Neurosci Ther.

[16] Keller T (2019). Intranasal breast milk for premature infants with severe intraventricular hemorrhage-an observation. Eur J Pediatr.

[17] Tarokh L (2011). Sleep EEG provides evidence that cortical changes persist into late adolescence. Sleep.

[18] Li J (2018). Structural covariance of gray matter volume in HIV vertically infected adolescents. Sci Rep.

[19] Xiao F (2019). Independent component analysis and graph theoretical analysis in patients with narcolepsy. Neurosci Bull.

[20] Chen X (2020). Topological abnormalities of functional brain network in early-stage Parkinson's disease patients with mild cognitive impairment. Front Neurosci.

[21] Hallett M (2020). Human brain connectivity: clinical applications for clinical neurophysiology. Clin Neurophysiol.

[22] Bullmore E, Sporns O (2009). Complex brain networks: graph theoretical analysis of structural and functional systems. Nat Rev Neurosci.

[23] Qin Y (2018). Functional connectivity alterations in children with spastic and Dyskinetic cerebral palsy. Neural Plast.

[24] Di Benedetto S (2019). Network topology dynamics of circulating biomarkers and cognitive performance in older Cytomegalovirus-seropositive or -seronegative men and women. Immun Ageing.

[25] Kim WSH (2022). Associations of white matter hyperintensities with networks of gray matter blood flow and volume in midlife adults: a coronary artery risk development in young adults magnetic resonance imaging substudy. Hum Brain Mapp.

[26] Fischer UM (2009). Pulmonary passage is a major obstacle for intravenous stem cell delivery: the pulmonary first-pass effect. Stem Cells Dev.

[27] Takasaki Y (2011). Estimation of the distribution of intravenously injected adipose tissue-derived stem cells labeled with quantum dots in mice organs through the determination of their metallic components by ICPMS. Anal Chem.

[28] Choi C (2018). The combination of mannitol and temozolomide increases the effectiveness of stem cell treatment in a chronic stroke model. Cytotherapy.

[29] Lochhead JJ, Thorne RG (2012). Intranasal delivery of biologics to the central nervous system. Adv Drug Deliv Rev.

[30] van Velthoven CT (2010). Nasal administration of stem cells: a promising novel route to treat neonatal ischemic brain damage. Pediatr Res.

[31] Torrado Pacheco A (2021). Sleep promotes downward firing rate homeostasis. Neuron.

[32] Vanderhaeghen P, Cheng HJ (2010). Guidance molecules in axon pruning and cell death. Cold Spring Harb Perspect Biol.

[33] Low LK, Cheng HJ (2006). Axon pruning: an essential step underlying the developmental plasticity of neuronal connections. Philos Trans R Soc Lond B Biol Sci.

[34] Riccomagno MM, Kolodkin AL (2015). Sculpting neural circuits by axon and dendrite pruning. Annu Rev Cell Dev Biol.

[35] Porkka-Heiskanen T (2013). Sleep homeostasis. Curr Opin Neurobiol.

[36] Tononi G, Cirelli C (2014). Sleep and the price of plasticity: from synaptic and cellular homeostasis to memory consolidation and integration. Neuron.

[37] Tan J (2014). Response of the sensorimotor cortex of cerebral palsy rats receiving transplantation of vascular endothelial growth factor 165-transfected neural stem cells. Neural Regen Res.

[38] Della Santina L (2021). Disassembly and rewiring of a mature converging excitatory circuit following injury. Cell Rep.

[39] Schuldiner O, Yaron A (2015). Mechanisms of developmental neurite pruning. Cell Mol Life Sci.

[40] Pfefferbaum A (1994). A quantitative magnetic resonance imaging study of changes in brain morphology from infancy to late adulthood. Arch Neurol.

