# Supplementary figures and images for: Safety and efficacy outcomes after intranasal administration of neural stem cells in cerebral palsy: a randomized phase 1/2 controlled trial
Source: Stem Cell Res Ther. 2023 Feb 9;14:23. doi: 10.1186/s13287-022-03234-y (PMC9910250; doi:10.1186/s13287-022-03234-y)

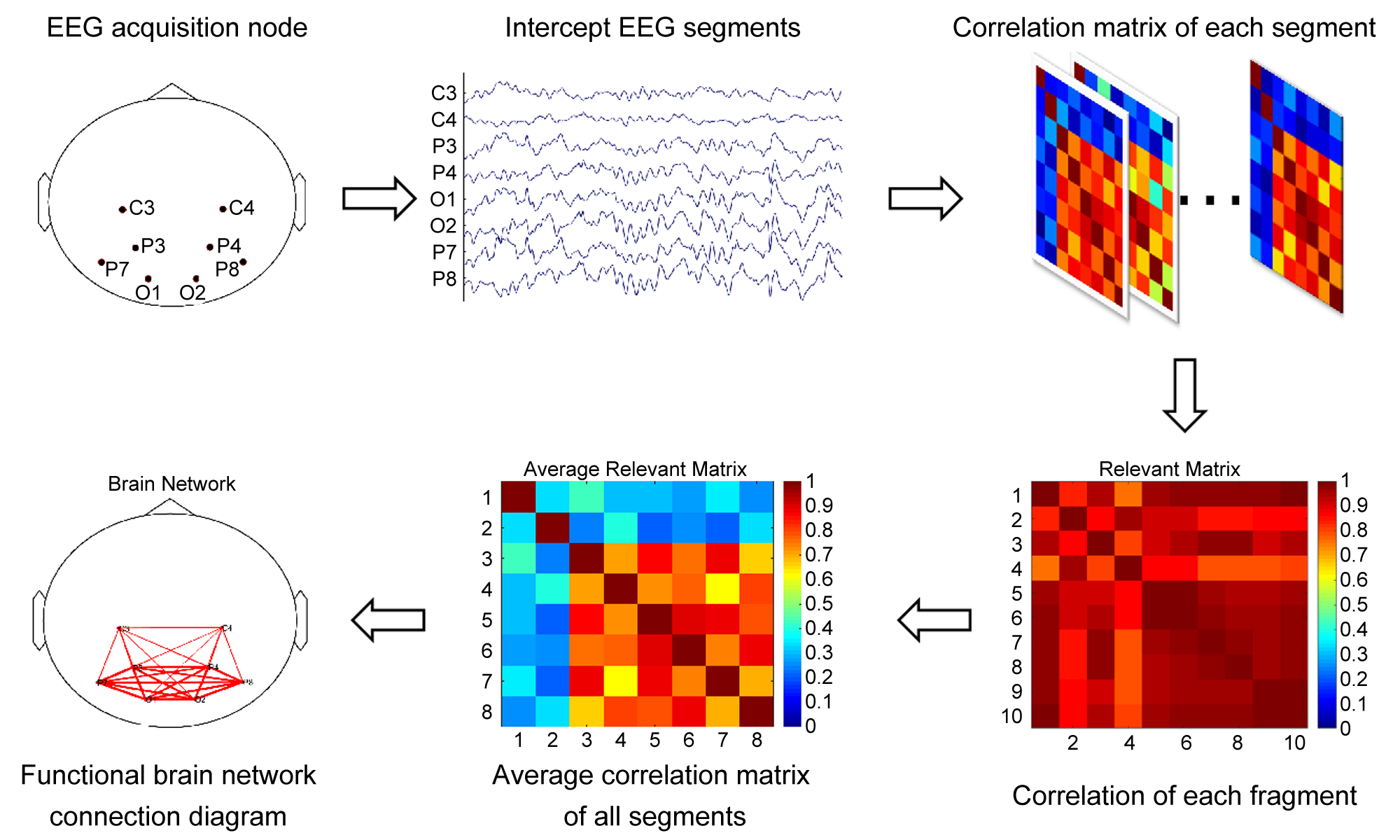

Supplement: Supplementary file 2 — Additional file 2. The EEG data processing flow. Red line: the Connectivity between Nodes; Node: the Locations of the 8 Electrodes (C3, C4, P3, P4, O1, O2, P7, P8). [file 13287_2022_3234_MOESM2_ESM.tif]

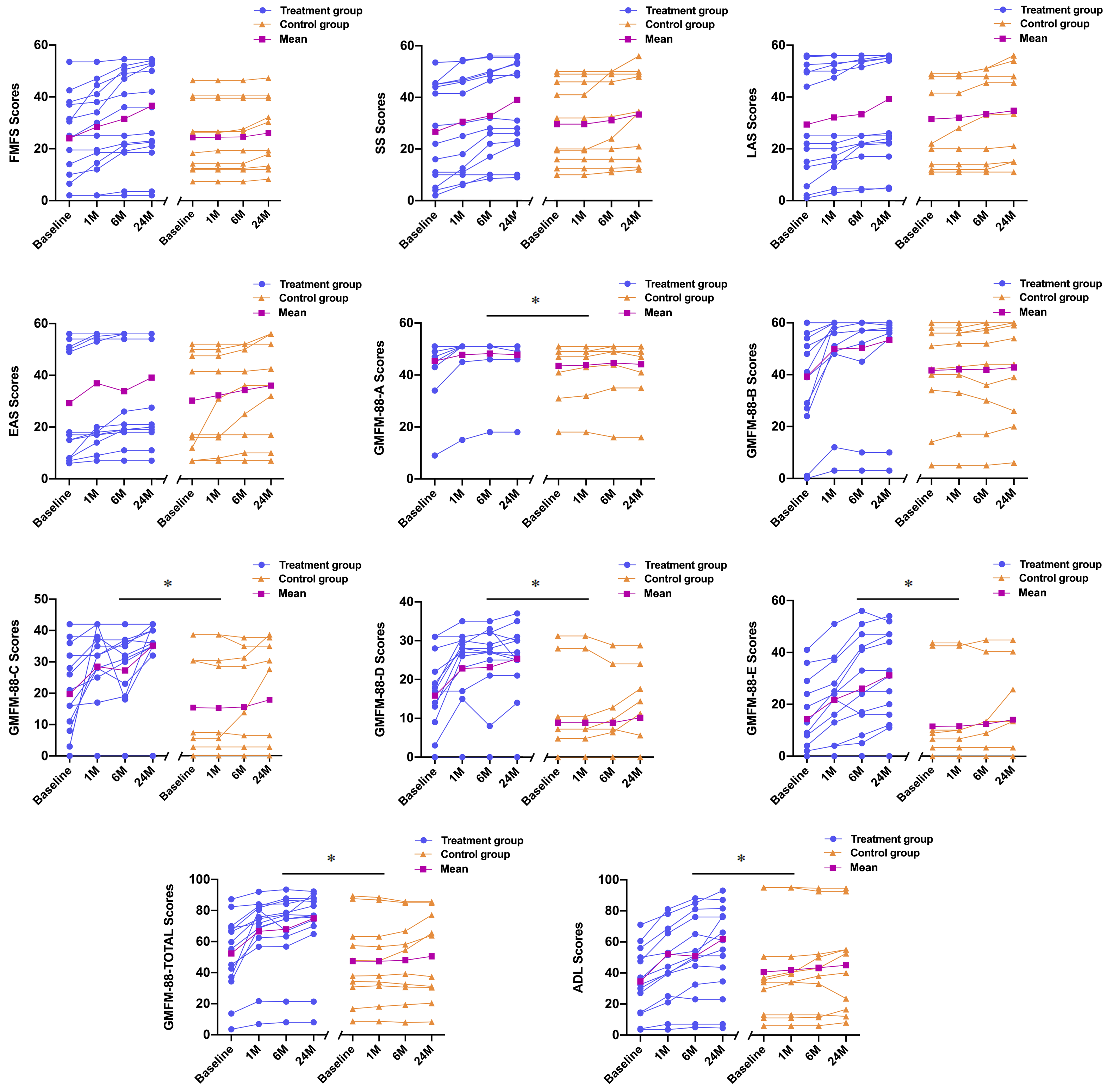

Supplement: Supplementary file 6 — Additional file 6. Changes of scales from baseline to month 24 between groups. 1M: 1 month after treatment; 3M: 3 months after treatment; 6M: 6 months after treatment; 24M: 24 months after treatment; GMFM: Gross Motor Function Measure; ADL: Activity of Daily Living; FMFS: Fine Motor Function Scale; SS: Sociability Scale; LAS: Life Adaptability Scale; EAS: Expressive Ability Scale; *: P<0.05. [file 13287_2022_3234_MOESM6_ESM.tif]
