# Supplementary material for: Safety and efficacy outcomes after intranasal administration of neural stem cells in cerebral palsy: a randomized phase 1/2 controlled trial
Source: Stem Cell Res Ther. 2023 Feb 9;14:23. doi: 10.1186/s13287-022-03234-y (PMC9910250; doi:10.1186/s13287-022-03234-y)
Supplement: Supplementary file 3 — Additional file 3. Functional brain network analysis methods. [file 13287_2022_3234_MOESM3_ESM.docx]

**Functional Brain Network Analysis Methods**

**1.** **Functional brain network construction**

A functional brain network (FBN) refers to a graph that includes the nodes and functional connections formed by the cooperation and coordination of neural activities between neurons and between parts of the nervous system. The method that is most commonly used to construct a FBN is the Pearson correlation coefficient (PCC). Assuming that the EEG signals acquired by the two electrodes are $x(t)$ and $y(t)$, t=1, 2 …. n, the correlation between the two can be expressed as follows:

$r=\frac{\sum_{i=1}^{n} \left( x_{i}-\bar{x} \right)\left( y_{i}-\bar{y} \right)}{\sqrt{\sum_{i=1}^{n} \left( x_{i}-\bar{x} \right)^{2}\sum_{i=1}^{n} \left( y_{i}-\bar{y} \right)^{2}}}$ (1)

In this formula, $r$ represents the PCC, and $x_{\mathcal{i}}$ and $y_{\mathcal{i}}$ correspond to the signal amplitudes of the x channel and y channel at the i-th moment, respectively. The larger A is, the stronger the correlation between $x(t)$ and $y(t)$; the smaller A is, the weaker the correlation between $x(t)$ and $y(t)$.

Preprocessed data was treated as a time-varying signal and the locations of the 8 electrodes (C3, C4, P3, P4, O1, O2, P7, P8) were used as nodes. The correlation was performed for the 8 channels data of 10 segments in each subject's sleeping state. The correlation coefficient between the two channels was used as the element of the matrix. Then, we obtained an 8th order adjacency matrix. Next, the correlation of 10 segments of data in each subject's awake or sleep state was calculated using PCC. If the correlation of the data for the 10 segments was high, then the average adjacency matrix of the 10 segments of data was used as the adjacency matrix in that subject's awake or sleep state. Finally, we used the average adjacency matrix to construct the functional brain network.

**2. Degree**

Degree of brain network is defined as the number of other nodes connected to the node. It reflects the importance of this node in the whole brain network. For undirected networks, the degree of nodes can be considered as the number of edges directly connected to node *i*:

$K_{i}=\sum_{j\in V} a_{ij}$ (2)

In this formula, $a_{ij}$ is the element of row *i* and column *j* in the adjacency matrix. The higher the degree of one node, the larger the nodes directly connected to this node, and the greater the impact of this node on the network. The average degree of all nodes in the network is called the average degree of the network, which can reflect the sparsity of edges in the network, or the total so-called "wiring cost" of the network.

**3. Clustering coefficient**

The clustering coefficient refers to the ratio between the actual number of edges between all neighbor nodes directly connected to the node *V(i)* and the maximum possible number of edges between these neighbor nodes. For the entire brain network $C(G)$, its value is equal to the average of the clustering coefficient $C(i)$ of each node and can be calculated by:

$C\left( G \right)=\frac{1}{N}\sum_{i\epsilon G} C\left( i \right)=\frac{1}{N}\sum_{(i,j,k)\epsilon G} \frac{2a(i,j)a(i,h)a(j,h)}{k_{i}(k_{i}-1)}$ (3)

The value of $C(i)$ is between [0, 1], and when the value is equal to 0, indicating that there is no connection between all neighbor nodes of node $v(i)$; a value that is equal to 1 indicates that there is a connection between node $v(i)$ and all the neighbor nodes. $G$ refers to the set of the entire network, $N$ refers to the number of network nodes, $C(i)$ represents the clustering coefficient of node$v(i)$, $a(i, j), a(i, h) and a(j, h)$ represents the elements of the adjacency matrix$. k_{i}$ represents the degree of node $v(i)$, and when $K_{i}< 3$, $C(i)= 0$.

**4. Characteristic path length**

The characteristic path length (*L*) of the FBN was defined as the average value of the paths between all nodes, which can be calculated as follows:

$L=\frac{1}{N(N-1)}\sum_{i,j\epsilon G,i\neq j} d(i,j)$ (4)

$L$ represents the entire set of the FBN, $N$represents the number of all nodes, $d(i,j)$ refers to the shortest path length between node $i$ and $j$, and was defined as the minimum number of connections from node $i$ and $j$. According to definition, $d(i,j)\geq1$. If $i$ and $j$ are neighbor node $d(i,j)=1$, if there is no connection between $i$ and $j$, $d(i,j)\to\infty$.

**5. Global efficiency**

Global efficiency measures the global transmission ability of the network; this is the inverse of all the shortest path lengths in a given network. When the set contained all nodes, the calculated efficiency represents the global efficiency.

$E_{global}\left( G \right)=\frac{1}{N\left( N-1 \right)}\sum_{i,j\epsilon G,i\neq j} \frac{1}{d\left( i,j \right)}$ (5)

$G$ represents the entire set of the network, $N$represents the number of all nodes, $d(i,j)$ represents the shortest path length. When $d(i, j)$→∞, $\varepsilon(i, j)$=0, there was no connection between nodes; when $d(i, j)$ =1, $\varepsilon(i, j)$ =1, the efficiency was the greatest.

**6. Brain network energy**

Brain network energy is an effective method with which to describe the topology of brain networks, which can be calculated by:

$E=\sum_{i=2}^{n} \sum_{j=1}^{i-1} r_{i,j}^{2}$ (6)

*N* represents the number of channels while *r* is the adjacency matrix.
